# Goose kidney organoids: A new model for studying the metabolic mechanism of goose astrovirus-induced gout

**DOI:** 10.1016/j.psj.2026.107500

**Published:** 2026-07-27

**Authors:** Yong Xiang, Linlin Li, Chenggang Liu, Junqin Zhang, Yunzhen Huang, Qi Zhai, Ming Liao, Minhua Sun, Jiawen Dong

**Affiliations:** a Institute of Animal Health, Guangdong Academy of Agricultural Sciences; Key Laboratory for Prevention and Control of Avian Influenza and Other Major Poultry Diseases, Ministry of Agriculture and Rural Affairs; Guangdong Provincial Key Laboratory of Livestock Disease Prevention, Guangzhou, Guangdong Province, PR China; b Shanwei Academy of Agricultural Sciences, Shanwei, Guangdong Province, PR China; c College of Animal Science & Technology, Zhongkai University of Agriculture and Engineering, Guangzhou, Guangdong Province, PR China

**Keywords:** Goose astrovirus, Kidney organoids, Infection model, Uric acid, Metabolic disturbance

## Abstract

Goose astrovirus (GAstV) is a newly identified avian pathogen that causes fatal gout in goslings, characterized by kidney damage and high mortality. Unlike non-pathogenic human gout, GAstV-induced gout lacks reference models due to the absence of goose-derived cell lines and standard experimental animals, limiting mechanistic studies. Here, primary goose kidney organoids (GKOs) were isolated from 28-day-old goose embryos. GKOs retained physiological structures and functions comparable to in vivo kidneys. GAstV infection in GKOs was evaluated across renal cell lineages and compared with in vivo outcomes. Viral replication and effects on uric acid metabolism were assessed at different multiplicities of infection. GAstV readily infected GKOs, targeting progenitor, pluripotent stem, collecting duct, and renal tubular cells, consistent with in vivo findings. At a multiplicity of infection of 10.0, GAstV rapidly induced a significant elevation of uric acid, whereas lower doses had weaker effects, paralleling disease severity in goslings. In contrast, GAstV replication was minimal in 2D cells, and other avian viruses failed to induce uric acid elevation in GKOs. Post-infection, transcriptomic and metabolomic analysis showed GAstV remodeled global gene expression and metabolism, mainly disrupting purine metabolism pathways. GAstV infection significantly increased mRNA levels of SLC22A23, SLC17A5, and SLC13A3, which were correlated positively with uric acid. GKOs provide a physiologically relevant model for elucidating GAstV pathogenesis, offering new insights into virus-induced gout and a platform for studying goose viral diseases.

## Introduction

Since 2016, a highly lethal gout disease in goslings caused by goose astrovirus (GAstV) has gradually appeared in geese in Anhui, Guangdong, Shandong, Hebei, and other provinces of China, eventually leading to a nationwide epidemic, indicating that the harm and pathogenicity of GAstV have increased significantly (Chen et al., 2020; Dong et al., 2026; Zhang et al., 2018, 2022). Different breeds of geese can be infected with GAstV, with an infection rate of up to 80%; however, adult geese are often uninfected (Li et al., 2025; Liu et al., 2023). It primarily affected goslings aged 1-20 days, with the most susceptible goslings being those less than 15 days old, resulting in a mortality rate of up to 50%. The main clinical symptoms are depression, loss of appetite, white stools, joint swelling, and unwillingness to walk (Xu et al., 2023, 2023). Pathological examination revealed swollen and pale kidneys, splenomegaly, and significant urate deposits on the surfaces of the heart, liver, and kidneys. Gout is a typical symptom that occurs in goslings following infection with GAstV (Fu et al., 2022; Song et al., 2026; Xiang et al., 2024). Therefore, it is considered a metabolic disorder. GAstV has been detected in the kidney, liver, spleen, lung, heart, and other tissues and organs, with the highest viral content in the kidney. Therefore, the kidneys are considered an important target organ for GAstV infection in geese (Liu et al., 2025; Yang et al., 2018; Zhang et al., 2018).

The LMH cell line is the most commonly used cell line for in vitro GAstV. It is derived from chicken, which is different from the host species of GAstV, and GAstV cannot replicate fully and efficiently in LMH cells. Therefore, it is more challenging to simulate related immune and metabolic responses after GAstV infection (Ren et al., 2020; Xiang et al., 2024). Goose embryo primary kidney cells (GEK) can proliferate in the presence of GAstV with high efficiency; however, it is often difficult and inconvenient to obtain GEK cells with high purity. Fibroblasts tend to become the dominant growth cells and gradually inhibit the growth of kidney cells, thus reducing the feasibility of subsequent studies and the scientific value of the data (Xiang et al., 2024). Goose embryo primary hepatocytes (GEH) face the same challenges More importantly, the in vitro cell model has a simple structure and a single cell type, which cannot simulate the three-dimensional structure and physiological functions of tissues and organs in vivo. Thus, it cannot respond appropriately to the metabolic responses of the organism. However, at present, there is no standard experimental animal for geese, which leads to the low repeatability of experimental results and large differences within groups, making it difficult to discover the underlying patterns. Up to now, due to the lack of an ideal research platform, the basic research on the pathogenic mechanism of GAstV is particularly slow, which greatly limits the scientific development of effective prevention and control strategies for highly fatal gouty goose gout caused by GAstV infection, and the prevention and control situation is still severe. Therefore, a more accurate infection model is needed to study the metabolism of GAstV, especially a kidney model, such as goose kidney organoids.

Although organoids have demonstrated their value as in vitro tools in the field of biomedicine, they are still in the early stages of development in veterinary science. Currently, only the intestinal organoids of chickens (Pierzchalska et al., 2019), and intestines (Li et al., 2019) and the liver tissues of pigs (Kruger et al., 2022) have been established. To date, there have been no reports on the use of organoids to study diseases in geese. Moreover, due to the significant pathogenic characteristics of GAstV, which primarily cause renal metabolic disorders and lead to urate deposition, there are higher requirements for cell models and platforms for studying GAstV, and kidney organoids can precisely meet these requirements.

In this study, we constructed goose kidney organoids (GKOs) derived from primary goose kidney cells and utilized this model to study GAstV infection. The results revealed that the GKOs were susceptible to GAstV infection and recapitulated many events associated with GAstV infection in the kidneys in vivo. Overall, these data indicate that GKOs can reproduce the key characteristics of in vivo kidneys, providing valuable resources for addressing the fundamental issues of GAstV, which cannot be simulated using traditional cell lines.

## Materials and methods

### Cells, viruses, and ethics statement

The LMH cells were cultured in DMEM/F12 (Gibco, Shanghai, China) and supplemented with 10% fetal bovine serum (TransGen, Beijing, China) at 37°C with 5% CO_2_. GEK and GEH are goose embryo primary kidney cells and goose embryo primary liver cells, respectively, which need to be prepared from 20-day-old goose embryo kidneys and 13-day-old goose embryo livers, which were cultured in DMEM (Gibco, Shanghai, China) and supplemented with 10% fetal bovine serum at 37°C with 5% CO_2_. The primary cell preparation method was based on a previously described procedure (Xiang et al., 2024). The GAstV GDYJ2210 strain was isolated from a goose with gout in Guangdong Province and maintained in our laboratory (Xiang et al., 2024). The Tembusu virus (TMUV) JM strain was isolated from diseased layer ducks in Guangdong province and maintained in our laboratory (Li et al., 2012), which also infects chickens and geese. The goose orthoreovirus (GRV) GD2020 strain was isolated from the liver and spleen of dead geese in Guangdong Province and maintained in our laboratory (Huang et al., 2022).

All animal experiments were performed in accordance with the regulation for animal experimentation of Guangdong Province, China, and were permitted by the Ethics Committee of Institute of Animal Health, Guangdong Academy of Agricultural Sciences (No. YC-PT2022040).

### Isolation of kidney cells and 3D culture of GKOs

The GKOs were prepared from 28-day-old embryonated goose eggs purchased from Chenyu Agricultural Products Co., Ltd. (Hengshui, China). Specifically, the embryo was incised under sterile conditions, and the kidney tissue was separated and placed in a phosphate-buffered saline (PBS) solution containing 1% penicillin and streptomycin for three rounds of washing via natural sedimentation of tissue fragments, with approximately 2 minutes of sedimentation each time. The kidney tissue was finely cut into small pieces, washed three times with PBS, and then discarded. The washed tissue fragments were treated with TrypLE™ Express Enzyme (Gibco, Shanghai, China) and subjected to digestion in a 37 °C water bath for 20 min. Add serum to terminate the digestion, and filter the supernatant through a 70 μm cell strainer (Sangon, Shanghai, China). The cells were then centrifuged for 5 minutes at 1000 rpm; the supernatant was discarded, and the cell sediment was retained. An appropriate amount of ACK Lysing Buffer (Gibco, Shanghai, China) was added and incubated at room temperature for 2 min to remove red blood cells. Centrifuge and retain the cell sediment. An appropriate amount of poultry kidney organoid culture medium (ORGEN, Beijing, China) was added, and the cell density was adjusted to approximately 400,000 cells/mL. The cell suspension and Matrigel (Mogengel, Xiamen, China) were mixed in a 3:7 ratio. Thereafter, 70 μL was taken and dropped onto a low-adhesion 24-well culture plate (Gibco, Shanghai, China). Incubation was performed at 37 °C for 30 minutes. After the glue droplet had solidified, 700 μL of organoid culture medium containing 10% goose serum was added around it and incubated at 37 °C with 5% CO_2_. The culture medium was replaced after 3 days, and cultivation was continued for an additional 9 days.

### Passaging and 2D culture of GKOs

The organoids were passaged for further cultivation when they reached maturity. The culture medium surrounding the matrix gel was removed. Pre-cooled PBS was added for one rinse to prevent damage to the Matrigel. Thereafter, 10 times the volume of the organoid harvesting solution (Mogengel, Xiamen, China) was added to the Matrigel. Finally, the mixture was transferred to a 15 mL centrifuge tube. The centrifuge tube was placed on ice and shaken for 60 min to allow the organoids to separate from the gel matrix. Centrifugation was performed at 4 °C for 5 minutes at 300 g, and the supernatant was discarded. An appropriate amount of TrypLE™ Express Enzyme (Gibco, Shanghai, China) was added to the sediment and incubated at 37 °C for 3 minutes. Subsequently, a small amount of fetal bovine serum was added to terminate the digestion. Centrifugation was performed at 300 × g for 5 min, the cell sediment was retained, and the previously described steps were followed to continue the 3D culture. The cell pellet was resuspended in the organoid medium and seeded into a 96-well plate. The cryopreservation and resuscitation of these organoids are the same as the operations for ordinary adherent cells; therefore, further details are not provided.

### Viral infection

A solution of the GAstV GDYJ2210 strain was added to the organoid culture medium, resulting in an approximate final multiplicity of infection (MOI) of 1.0. Notably, unlike conventional 2D monolayer cells, organoids preclude accurate cell counting. Thus, MOI values herein are approximations calculated from the initial cells seeded for 3D culture. Samples were collected 48 h after viral infection for hematoxylin and eosin (HE) staining, immunohistochemistry, and frozen section immunofluorescence analysis. On the 1st, 2nd, 3rd, 5th, and 7th days after GAstV infection, pathological changes in GKOs mice were observed under a microscope. To analyze the effect of GAstV replication on uric acid, creatinine, and nitrogen levels, GKOs, LMH, GEK, and GEH cells were infected with the virus at MOIs of 0.1, 1.0, and 10.0, respectively. Samples were collected 24, 48, 72, and 96 h after viral infection for detection and analysis. To clarify whether GAstV is the main factor causing a significant increase in uric acid levels in GKOs, GAstV, TMUV, and GRV were used to infect GKOs at an MOI of 10.0. Samples were collected 24, 48, 72, and 96 h after viral infection, and uric acid levels were measured. In addition, GKOs, LMH, GEK, and GEH cells were infected with GAstV at an MOI of 1.0, and the differences in GAstV replication levels were analyzed using reverse transcription quantitative PCR (RT-qPCR).

### Collection of animal tissue samples

During an initial epidemiological investigation of GAstV in Guangdong Province, our team collected a large number of tissue samples that were naturally infected with GAstV and exhibited varying degrees of clinical symptoms (Liu et al., 2023; Xiang et al., 2024). In this study, to fully demonstrate the scientific validity of GKOs, we conducted a comparative analysis of some animal tissue samples, including goose kidney tissue samples from cases where GAstV infection resulted in severe gout (near death), mild gout, and asymptomatic conditions.

### HE staining

HE staining was performed on the GKOs and animal kidney tissue samples. First, the samples were fixed in 4% paraformaldehyde and then embedded in paraffin and sectioned at a thickness of 5 μm. Sections were mounted on slides, deparaffinized with xylene, and rehydrated using a graded alcohol series. The sections were stained with hematoxylin for 10 min, rinsed, differentiated, and stained blue with tap water. The sections were counterstained with eosin for 2 min, dehydrated with alcohol, cleared in xylene, and mounted on coverslips for microscopic examination.

### Immunohistochemical (IHC)

For IHC staining of GAstV VP27 protein in animal tissues and organoid samples, fixation, embedding and sectioning were performed as described above. The sections were deparaffinized with xylene and rehydrated using a graded alcohol series, followed by antigen retrieval using citrate buffer (pH 6.0) via heat-induced epitope retrieval. Endogenous peroxidase was blocked with 3% H₂O₂, and non-specific binding was blocked with 10% normal serum. The sections were then incubated overnight at 4°C with rabbit polyclonal primary anti-VP27 antibody (1:1200 dilution), which was previously prepared and stored in our laboratory (Xiang et al., 2024). The HRP-conjugated secondary antibody was applied for 360 minutes at room temperature. The signal was developed using a diaminobenzidine substrate, counterstained with hematoxylin, dehydrated, cleared in xylene, and mounted on coverslips for microscopic analysis.

### Frozen section immunofluorescence

To confirm whether the cultured organoids were renal, immunofluorescence staining was performed for various cell lineage marker proteins in the kidney. The processing method for the preliminary samples was based on immunohistochemistry, utilizing antibodies specific to the corresponding target protein during the staining process. This study included renal progenitor cell markers (anti-SALL1 antibodies, Cat. ab31526; Abcam, and anti-SIX2, Cat. sc-398193, Santa Cruz Biotechnology, Shanghai, China), pluripotent stem cell markers (anti-LGR5, Cat. DF2816, Affinity, Liyang, China), tube cell markers (anti-PAX2, Cat. sc-514352, Santa Cruz Biotechnology, Shanghai, China, and anti-GATA3, Cat. AF6233; Affinity, Liyang, China), remote tubules, and the collecting duct marker (anti-E-Cad, Cat. AF0131, Affinity, Liyang, China). The usage method of the abovementioned antibodies was in accordance with the manufacturer’s instructions. The selection of these markers was based on relevant published literature(Barker et al., 2012; Morizane et al., 2015; Takasato et al., 2015). In the subsequent analysis of GAstV infection localization in GKOs, the VP27 antibody was also used following the same procedure as described above. The markers of each renal cell lineage were double-stained with VP27 for fluorescence examination. Furthermore, this analysis was applied to animal tissue samples to examine the consistency between organoids and those found in animal bodies. The primary antibody was incubated overnight at 4 °C and washed. The fluorescent secondary antibody was incubated at 37 °C for 15 minutes, including anti-Alexa Fluor 647-labeled goat anti-mouse/rabbit IgG (H+L, A0473/A0468, Beyotime, Shanghai, China) for cell marker, Alexa Fluor 488-labeled goat anti-rabbit IgG (H+L, A0423, Beyotime, Shanghai, China) for VP27.When the two target antibodies used in the double-labeling staining are both from the same species, the multiplex sequential immunostaining technique, based on tyramine signal amplification, can be employed to label the two different target proteins successively. DAPI (4′,6-diamidino-2-phenylindole, Beyotime, Shanghai, China) was used to stain cellular nuclei. Store at 4 °C and protect from light. The stained cells were visualized under a fluorescence microscope.

### Determination of uric acid, creatinine, and urea nitrogen

The excessive accumulation of uric acid causes the occurrence of gout; therefore, uric acid levels are a key indicator for evaluating gout. Transfer 2D cultured GKOs, LMH, GEK, or GEH cells from a 96-well plate to a centrifuge tube and centrifuge to retain the cell pellet. Subsequently, 200 μL of physiological saline was added to each sample, and the samples were homogenized by grinding using a frozen tissue grinder. The supernatant was collected by centrifugation at 1000 r/min for 10 minutes at 4°C and used for the detection of uric acid and creatinine. Specific operative procedures were performed according to the manufacturer’s recommendations for uric acid (Cat. ml076336), creatinine (Cat. M12C4L), and urea nitrogen (Cat. ml076479) (Enzyme-Linked Biotechnology Co., Ltd., Shanghai, China). For the determination of animal tissue samples, approximately 0.1 g of tissue is weighed from each sample, and 1 mL of physiological saline is added for ice bath homogenization. The supernatant was collected for detection by centrifugation, as described above.

### RT-qPCR

Total RNA was extracted using an RNA extraction kit (Fastagen, Shanghai, China), according to the manufacturer’s recommendations. First-strand cDNA was synthesized from 500 ng of total RNA as a template, along with random primers, using the PrimeScript RT Reagent kit (TaKaRa, Beijing, China). Then, RT-qPCR was performed employing Hieff® qPCR SYBR Green Master Mix (Yeasen, Shanghai, China) on a Roche LightCycler96 System (Roche Diagnostics, Basel, Switzerland). Tissue samples, approximately 0.1 g of tissue, were weighed for RNA extraction. GAstV virus replication was determined using RT-qPCR assay and expressed as virus RNA copies/μL(Liu et al., 2023). The gene relative expression levels of solute carrier family 22 member 23 (SLC22A23), solute carrier family 13 member 3 (SLC13A3) and solute carrier family 17 member 5 (SLC17A5) were quantified using the 2^−ΔΔCt^ method and normalized to GAPDH expression. The primers used in this study are listed in Table 1.

**Table 1:** Reverse transcription quantitative PCR primers used in this study.

Primer^a^	Sequence (5′–3′)	GenBank
GAstV -F	GGGTGATCCGCAAGGAAATA	OP776629.1
GAstV -R	AAGTTTCGCCAGGGTTAGAG	
SLC22A23-F	CGAGGCGAAGATGACGATCA	XM_048050208.2
SLC22A23-R	CTCCTCCCCAGAAACCCAAC	
SLC17A5-F	AGGGAGTAAGCAAGGTGA	XM_066994669.1
SLC17A5-R	CTGATACGCAGGGATGGA	
SLC13A3-F	TTTGGGGAGAAAGACGCCTC	XM_066978346
SLC13A3-R	CCTCCTTCTCCTCCGTCAGA	
GAPDH-F	CTGTCAAGGCTGAGAATG	AY436595.1
GAPDH-R	CAAGAGGCATTGCTGACA	

a Forward and reverse primers are indicated by F and R, respectively.

### Transcriptomic and untargeted metabolomic analysis

We speculate that viral infection may regulate one or several key switch molecules in kidney cells, thereby inducing metabolic disorders and obstructing uric acid excretion, which ultimately leads to elevated uric acid levels. To screen these pivotal molecules, samples were collected from virus-infected GKOs (MOI=10.0) at 96 hours post-infection and sent to Personalbio Technology Co., Ltd. (Shanghai, China) for transcriptome sequencing, untargeted metabolomic analysis, and integrated multi-omics joint analysis. Total RNA was extracted for transcriptome sequencing. cDNA libraries were constructed and sequenced on high-throughput platform. Clean reads were mapped to reference genome, followed by gene expression quantification and differential expression analysis. For untargeted metabolomics, samples were prepared and detected via liquid chromatography-tandem mass spectrometry (LC-MS). Metabolite peaks were identified, quantified and annotated. Differential metabolites were screened for functional enrichment and pathway analysis.

### Statistical analysis

Data were analyzed using Prism software (GraphPad Prism 9; La Jolla, CA, USA). Statistical tests were performed using Student’s t-test for one-group and two-group comparisons, and one-way analysis of variance (ANOVA) with Bonferroni’s post-test for multiple-group comparisons. A p-value of less than 0.05 was considered significant (*p < 0.05, ** p < 0.01, *** p < 0.001, **** p < 0.0001).

## Results

### The generation of GKOs

**Fig. 1:**
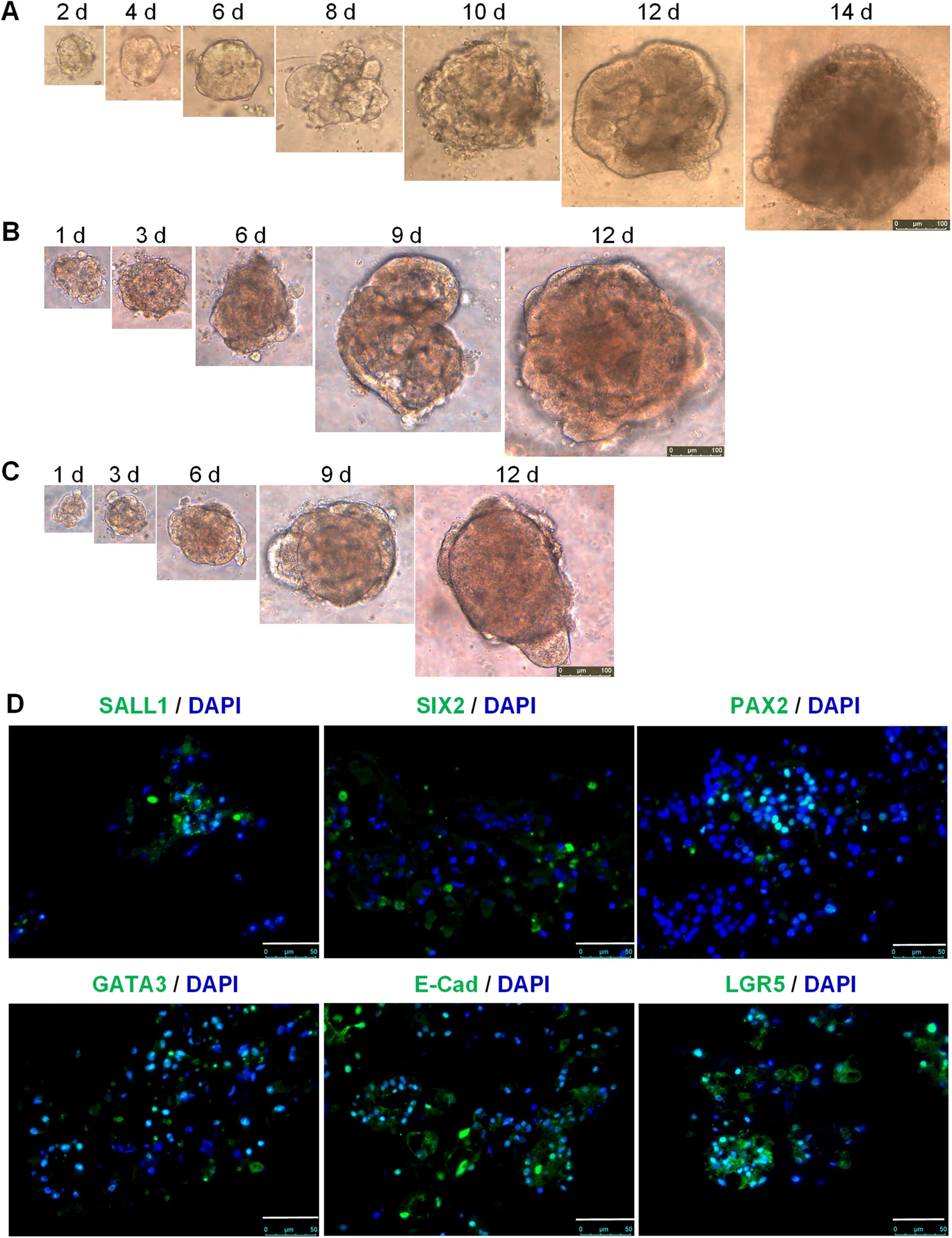
The cultivation and identification of GKOs.

To determine the optimal age of goose embryos for preparing primary GKOs, we compared the effects of 20-day-old and 28-day-old embryos, as well as 1-day-old goslings after hatching (Supplementary Fig. S1). Preliminary results showed that the best outcome was achieved when 28-day-old goose embryos were used to prepare the GKOs. After one week of cultivation, both the number and size of the organoids significantly increased. Fig. 1**A** shows the growth status of GKOs cells formed from primary kidney cells after different periods of culture. It can be observed that on the second day of cultivation, the rudimentary form of organoids began to appear, with clear contours and smooth edges; subsequently, they gradually grew, differentiated and enlarged, and their color gradually deepened. At this point, mature organoids were subcultured for further cultivation. They exhibited robust growth, with a rate slightly exceeding that of the primary cultures (Fig. 1**B**). Furthermore, the cells continued to grow well after cryopreservation, thawing, and reculturing (Fig. 1**C**). In addition, the results of immunofluorescence staining showed that the markers of each renal cell lineage were all positive, suggesting that this type of organ is composed of various cells, such as pluripotent stem cells, renal progenitor cells, distal tubules, and collecting duct systems, which is consistent with the physiological characteristics of the kidney. This indicated that goose kidney organoids were successfully cultivated (Fig. 1**D**). These data indicate that the GKOs were successfully cultivated.

**(A)** Primary culture of kidney primary cells to generate GKOs, passage culture of mature GKOs **(B)**, and cryopreservation and resuscitation culture **(C),** Scale bars: 100 μm; **(D)** Immunofluorescence staining for identifying the markers of each cell lineage in GKOs. Includes renal progenitor cell markers targeting anti-SALL1 and anti-SIX2, pluripotent stem cell markers targeting anti-LGR5, collecting tube cell markers anti-PAX2 and anti-GATA3, remote tubule and collecting duct markers anti-E-Cad. The cell marker protein is green and the cell nucleus is blue, Scale bars: 50 μm.

### GKOs are susceptible to GAstV infection

**Fig. 2:**
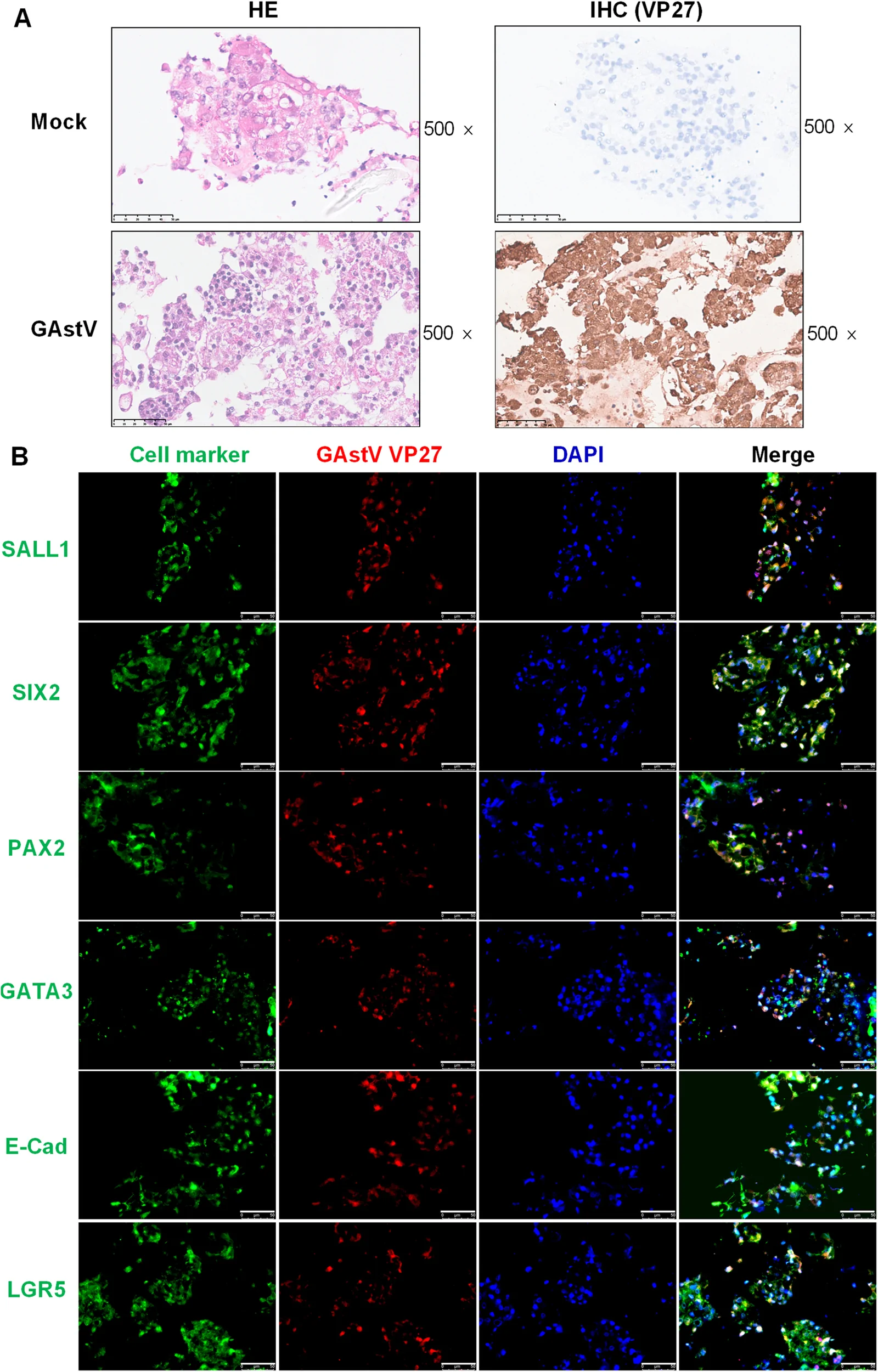
Phenotypic identification of GAstV-infected GKOs *in vitro.*

To determine whether GAstV can infect GKOs, we first conducted IHC analysis of GAstV VP27 and then analyzed the pathological changes after the virus was infected using HE staining. The results showed that GKOs exhibited the structural characteristics of renal tissue in vivo, and IHC analysis revealed that VP27-positive cells almost completely covered the area, suggesting that GAstV can infect various kidney structures. After GKOs were infected with GAstV, a large number of cells showed necrosis and detachment, the arrangement of renal cells was disordered (Fig. 2**A**). To further clarify which cell lineages were infected with GAstV in GKOs, we conducted an analysis using dual immunofluorescence labeling. The results showed that GAstV co-localized with SALL1- and SIX2-positive renal progenitor cells, LGR5-positive pluripotent stem cells, PAX2- and GATA3-positive collecting duct cells, and E-positive distal tubules (Fig. 2**B**), indicating that GAstV can infect multiple cell lineages in GKOs.

**(A)** HE staining and IHC staining of VP27 in GKOs with GAstV infection. Scale bars: 50 μm. **(B)** Dual fluorescence immunostaining of GAstV VP27 and the markers of each cell lineage of GKOs. Includes renal progenitor cell markers targeting anti-SALL1 and anti-SIX2, pluripotent stem cell markers targeting anti-LGR5, collecting tube cell markers anti-PAX2 and anti-GATA3, remote tubule and collecting duct markers anti-E-Cad. The cell marker protein is green and the cell nucleus is blue, Scale bars: 50 μm.

**Fig. 3:**
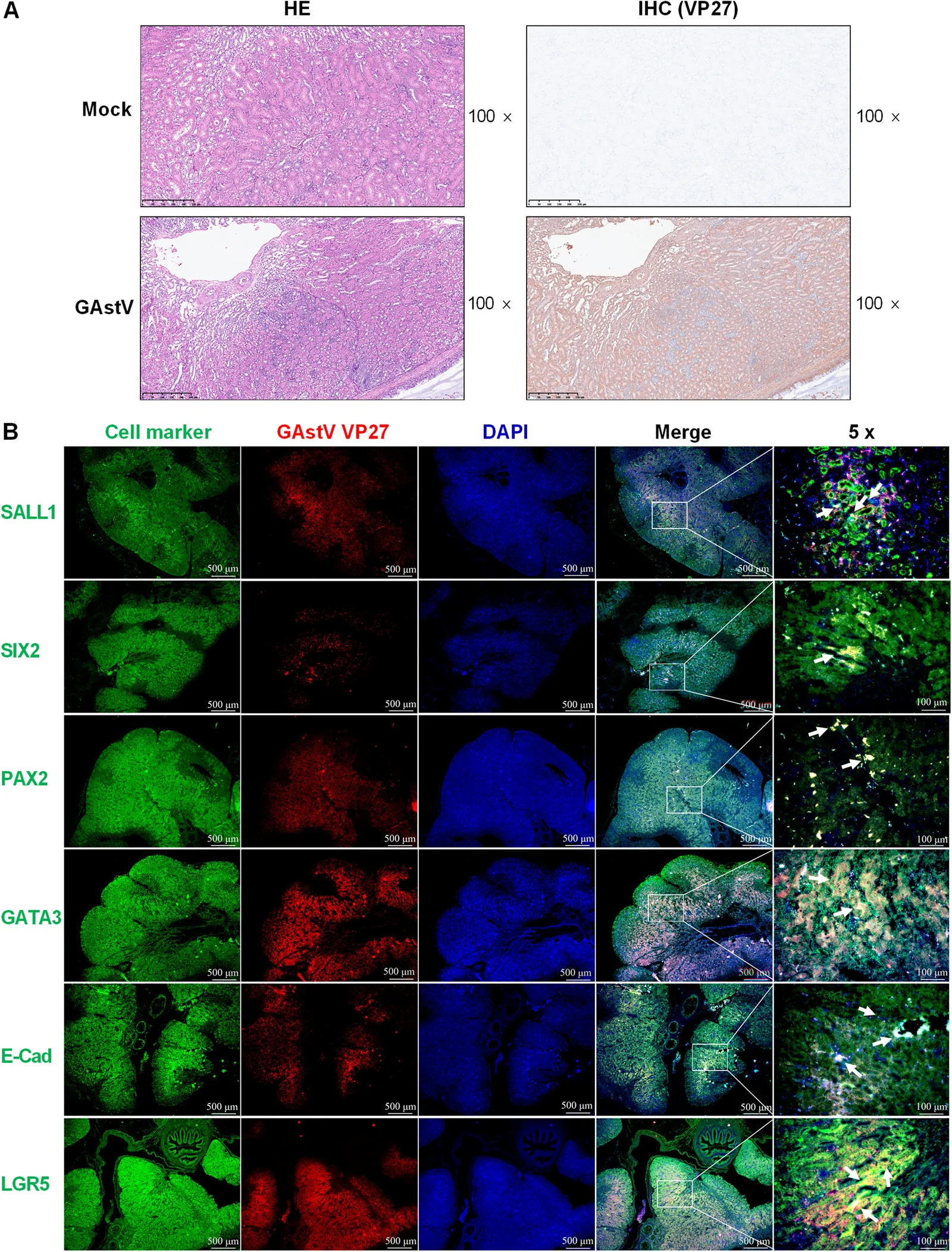
Phenotypic identification of GAstV-infected kidney tissue *in vivo.*

To further verify whether GAstV infects various cell lineages in goose kidneys and the pathological changes caused by infection *in vivo*, we conducted IHC analysis for VP27, HE staining, and dual immunofluorescence staining of the collected goose kidney tissues. The data show that in kidneys infected with GAstV, a large number of epithelial cells of the renal tubules are detaching, the arrangement of renal cells is disordered, and the cell nuclei are shrinking. IHC results showed that VP27-positive cells also covered almost the entire area (Fig. 3**A**), which is similar to those in GKOs. Furthermore, GAstV can colonize multiple cell lineages in various organs such as the renal tubules, collecting ducts, and distal tubules (Fig. 3**B**). These data indicate that the in vitro infection of GKOs by GAstV is similar to the in vivo infection of goose kidneys. Thus, GKOs have established a unique platform for in-depth and efficient exploration of multiple cellular targets and pathogenic mechanisms of GAstV.

**(A)** HE staining and IHC staining of VP27 in kidney tissue with GAstV infection. Scale bars: 250 μm. **(B)** Dual fluorescence immunostaining of GAstV VP27 and the markers of each cell lineage of kidney tissue. Includes renal progenitor cell markers targeting anti-SALL1 and anti-SIX2, pluripotent stem cell markers targeting anti-LGR5, collecting tube cell markers anti-PAX2 and anti-GATA3, remote tubule and collecting duct markers anti-E-Cad. The cell marker protein is green and the cell nucleus is blue, Scale bars: 500 μm.

### GAstV infection causes significant pathological changes in GKOs

**Fig. 4:**
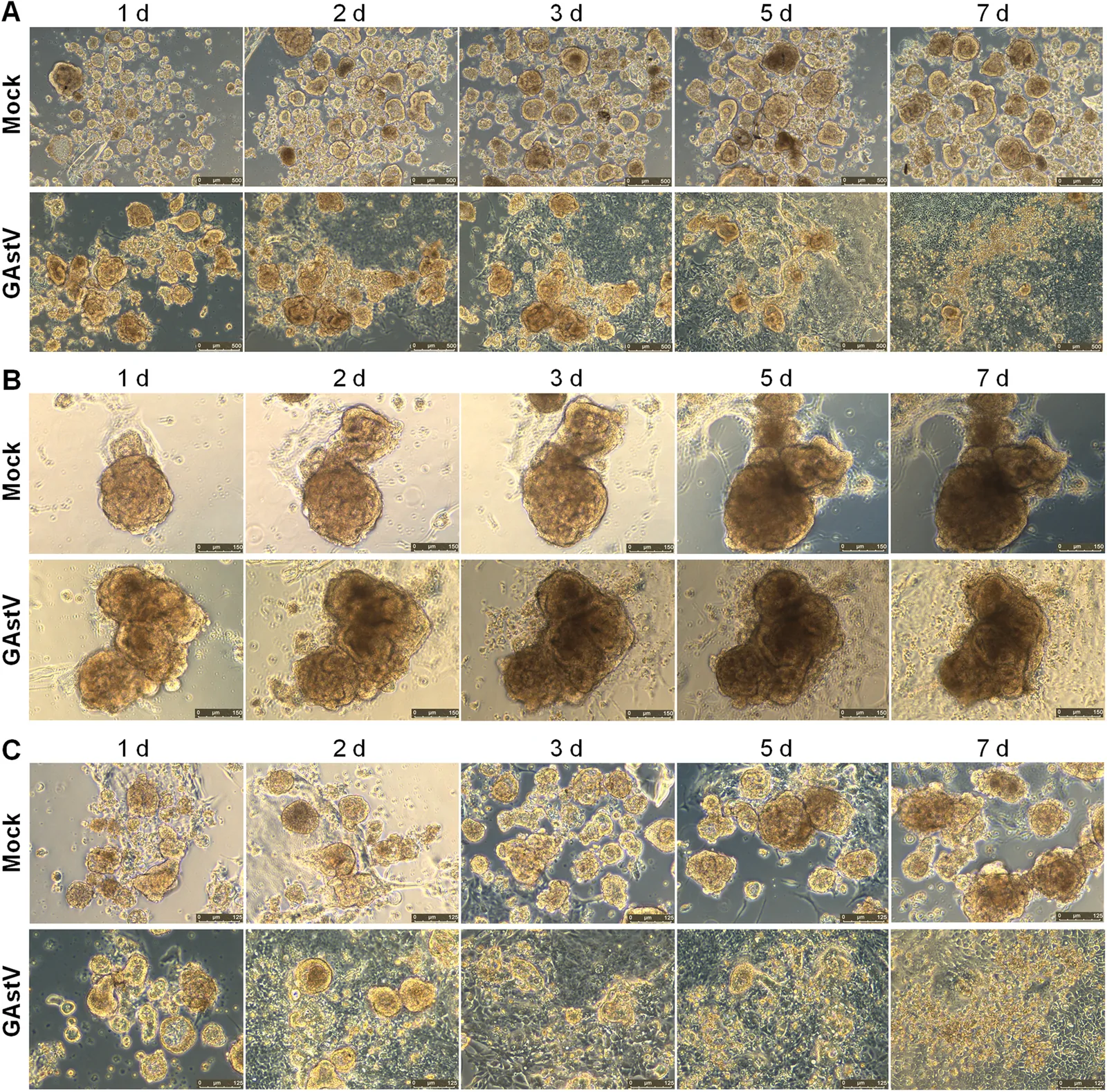
The pathological characteristics caused by GAstV infection in GKOs.

This study also clarified the characteristics of the pathological changes that occur in GKOs owing to GAstV infection. When GAstV-infected GKOs were at an MOI of 1.0, small organoids began to break, disappear, and die on the 2nd day after infection. Subsequently, the number of disappearing organoids increased daily. Many epithelial or fibroblast cells were generated in the areas where the vanished organoids were located and around them (Fig. 4**A**). Larger and more mature organoid spheres seem to have a greater tolerance to GAstV and are less likely to be damaged. To clarify this point, we separated the class organ spheres of different sizes by static sedimentation, and then cultivated and infected them with viruses. The results showed that for large and mature individual organs, when the virus attacks them for 7 days, the damage caused is quite limited (Fig. 4**B**). However, in smaller and less mature organoids, the damage caused by GAstV is obvious and extremely severe. Seven days after the viral infection, all patients had completely collapsed. There were no visible organoids, and everywhere one could see were merely epithelial cells or fibroblast cells (Fig. 4**C**). Based on these characteristics, GKOs can provide a superior platform for screening antiviral drugs.

**(A)** The overall pathological characteristics of GAstV infection in various growth stages of GKOs. Scale bars: 500 μm. **(B)** GAstV has a limited impact on large and mature organoids with a relatively large volume. Scale bars: 150 μm. **(C)** GAstV has a very significant destructive effect on small and immature organoid spheres. Scale bars: 125 μm.

### High-level replication of GAstV induces high uric acid levels in GKOs, consistent with the in vivo findings

**Fig. 5:**
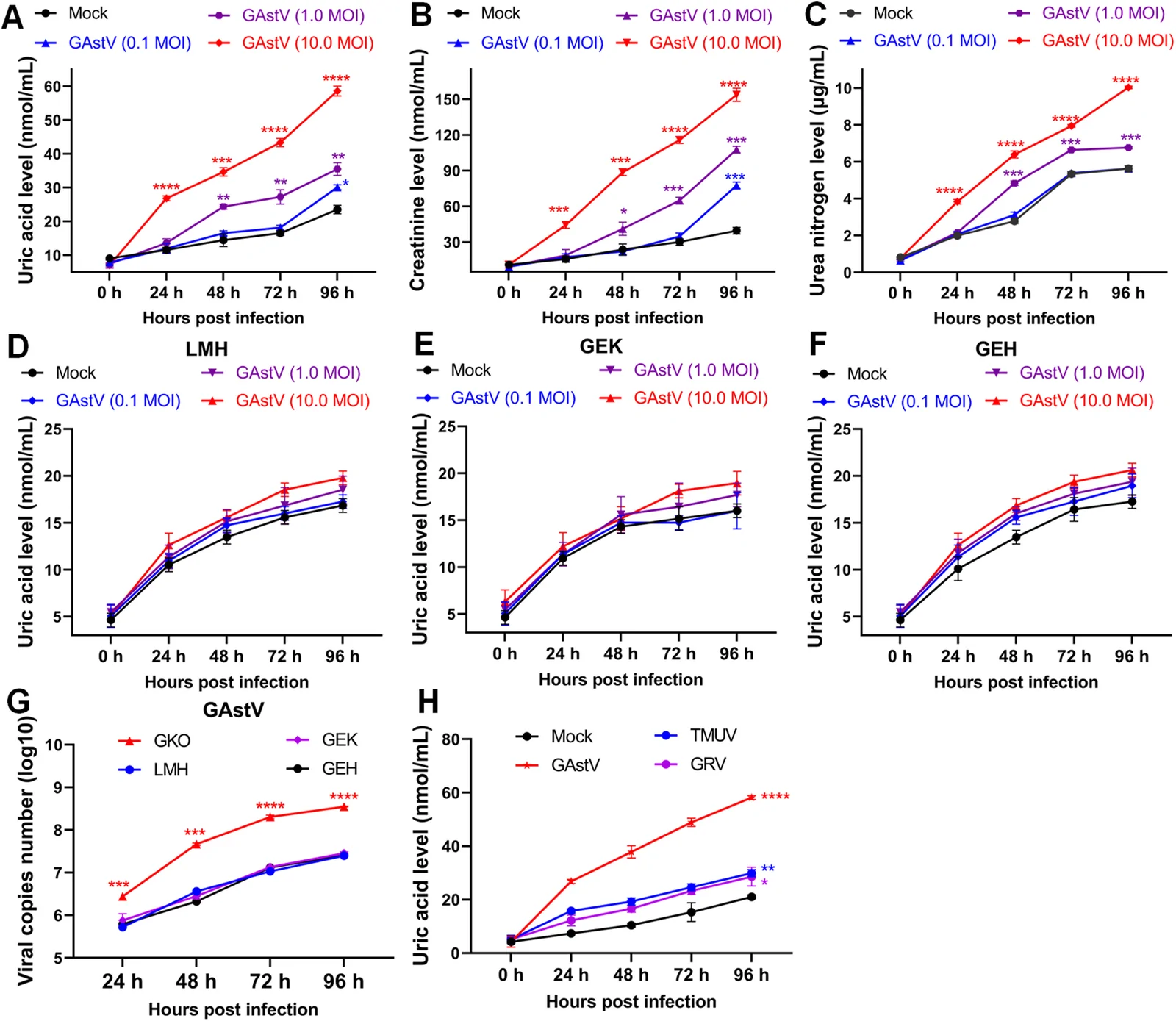
High-level replication of GAstV induces high uric acid levels in GKOs.

Gout, resulting from elevated uric acid levels, represents the primary pathological outcome of GAstV infection in young geese. To assess the relationship between viral replication and uric acid accumulation, GKOs were infected with varying viral titers, and their response was evaluated to determine the suitability of GKOs as a platform for investigating the metabolic mechanisms underlying GAstV-induced gout. The results showed that when GKOs were infected with 10.0 MOI of GAstV, high uric acid levels were induced after 24 h, and the uric acid level continued to increase, which was significantly more pronounced than at 0.1 and 1.0 MOI. When GAstV were infected with GKOs at an MOI of 0.1 and 1.0, it could only induce high uric acid levels in the later stage of infection. Although it was significantly higher than that of the control group without infection, there was still a gap compared to the MOI of 10.0 (Fig. 5**A**). At the same time, we also measured the levels of creatinine and uric acid nitrogen in the GKOs. These results are consistent with the trend of changes in uric acid levels. After viral infection at an MOI of 10.0, the levels of creatinine and urea nitrogen increased rapidly and continuously, indicating significant impairment of renal function, which was significantly higher than that in the 1.0 MOI group. In contrast, there was no significant difference between the 0.1 MOI infection group and the blank control group (Fig. 5**B-C**). This suggests that highly replicating viruses can cause significant damage to the kidneys, thereby facilitating the rapid production of uric acid.

GKOs were infected with GAstV at MOI values of 0.1, 1.0 and 10.0 respectively, and the changes in their uric acid **(A)**, creatinine **(B)** and urea nitrogen levels **(C)** were monitored. In the same way, GAstV was respectively infected into LMH **(D)**, GEK **(E)** and GEH **(F)** cells, and the changes in their uric acid levels were detected. **(G)** The replication levels of GAstV in GKOs, LMH, GEH and GEK cells differed, and were presented in terms of virus copy number. **(H)**The effects of different viral infections (10.0 MOI) on the uric acid levels in GKOs. *p < 0.05, ** p < 0.01, *** p < 0.001, **** p < 0.0001.

However, when we infected these 2D cell lines (LMH, GEK, and GEH) with the virus in the same manner, even at a high-titer infection dose, it was impossible to induce significantly high uric acid levels. Although uric acid levels also increased, there was no difference compared to the blank control group, and the absolute levels of uric acid were significantly lower than those when the animals were infected with GKOs (Fig. 5**D-F**). This indicates that GKOs can serve as an ideal platform for inducing hyperuricemia in GAstV models, providing a solid foundation for the in-depth exploration of the pathogenic mechanism of GAstV. This cannot be achieved using conventional 2D cells. What are the reasons for these discrepancies? In addition to the unique structural and physiological advantages of organoids, are there any differences in the viral replication levels in GKOs and 2D cells? The results showed that the replication level of GAstV in GKOs cells was significantly higher than that in the 2D cells (Fig. 5**G**).

Finally, to confirm that the induction of high uric acid levels by the virus is a unique characteristic of GAstV, we infected GKOs with the common waterfowl viruses TMUV and GRV, as well as GAstV, at an MOI of 10.0, and monitored the changes in uric acid levels. The results showed that the high uric acid levels induced by GAstV were significantly higher than those caused by TMUV and GRV, with a substantial difference. Compared to the blank control group, TMUV and GRV also significantly promoted uric acid production; however, the absolute differences were not significant and were much lower than those induced by GAstV (Fig. 5**H**). These data suggest that the primary factor causing gout in goslings remains GAstV, and a high level of viral replication has a positive effect on the occurrence of gout.

In addition, we compared uric acid levels and GAstV viral loads between the organoid infection model and kidney tissues from gout-infected goslings at different disease stages. The collected goose kidney tissues were classified into four groups based on clinical symptoms and pathological changes in the kidneys after autopsy: healthy control, asymptomatic infection, mild gout, and severe gout (near death). The results showed that kidney tissues with severe gout had the highest levels of uric acid and GAstV viral load, followed by those with mild gout. The uric acid levels in the asymptomatic group were not significantly different from those in the control group (Supplementary Fig. S2). This is consistent with the conclusion drawn by GKOs, which indicates that the occurrence of gout is closely related to the efficient replication of the virus. This further confirmed the validity and reliability of the organoid model, which exhibited high consistency with in vivo results.

### Global metabolome and transcriptome changes induced by GAstV infection in GKOs

Untargeted metabolomic differential analysis revealed that a total of 475 differential metabolites were identified in the GAstV-infected group relative to the control group, including 402 upregulated metabolites and 73 downregulated metabolites (Supplementary Fig. S3A-B). As indicated by the heatmap of differential metabolites, samples from the control group and GAstV-infected group were distinctly clustered separately, suggesting that GAstV infection induced global reprogramming of the metabolic profile. The altered metabolites were involved not only in lipid metabolism and amino acid/peptide metabolism but also in amine metabolism, implying that GAstV may regulate cellular functions synergistically via multiple metabolic pathways (Supplementary Fig. S3C-D). Kyoto encyclopedia of genes and genomes (KEGG) enrichment results showed that the significantly enriched pathways were primarily associated with purine metabolism, uric acid biosynthesis and their downstream metabolic processes. Purine metabolism stood out as the most prominent pathway, and pathways linked to purine synthetic precursors as well as energy, carbon and nitrogen metabolism were also significantly enriched (Supplementary Fig. S3E). Metabolites involved in the core pathways are listed in Supplementary Table S1. Meanwhile, metabolite abundance profiles revealed that GAstV infection significantly increased the intracellular levels of uric acid, creatinine and urea nitrogen (Supplementary Fig. S3F).

Transcriptomic analysis identified a total of approximately 1327 significantly differentially expressed genes in the GAstV-infected group, consisting of 760 downregulated genes and 667 upregulated genes (Supplementary Fig. S4A-B). This finding indicated that GAstV infection triggers extensive reprogramming of the global transcriptional program. Gene ontology (GO) enrichment results showed that the significantly enriched functional categories were mainly assigned to biological processes and cellular components associated with immune and inflammatory responses, receptor-mediated signal transduction, and extracellular microenvironment remodeling. In terms of molecular functions, enriched terms were closely related to ion binding and cytokine activity (Supplementary Fig. S4C). KEGG enrichment results showed that enriched signaling pathways were predominantly involved in cell communication, inflammatory cytokine regulation, and cytoskeletal and adhesion remodeling. Among them, Neuroactive ligand–receptor interaction and Cytokine–cytokine receptor interaction were the most prominently enriched pathways. In addition, obvious enrichment signals were also observed in metabolism-related pathways, such as linoleic acid metabolism, arachidonic acid metabolism and arginine biosynthesis. These results suggested that amino acid and lipid metabolism may be closely linked to the production of inflammatory mediators and alterations in uric acid homeostasis. Specifically, the synthesis of inflammation-related lipid mediators could modulate metabolic processes and immune responses (Supplementary Fig. S4D).

### SLC22A23, SLC17A5 and SLC13A3 serve as potential core factors mediating GAstV-induced uric acid elevation

**Fig. 6:**
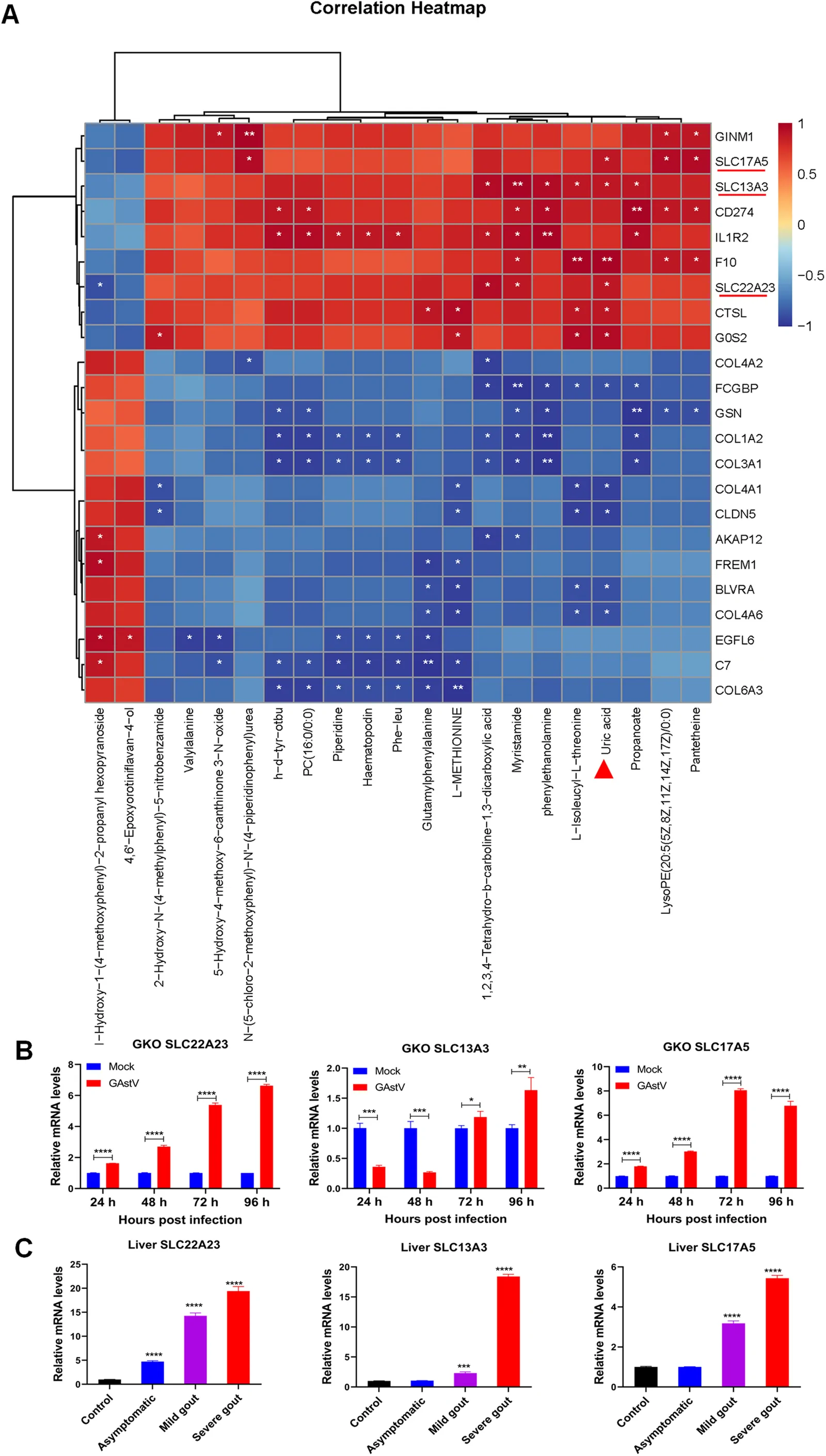
**SLC22A23, SLC17A5 and SLC13A3 serve as potential core factors mediating GAstV-induced uric acid elevation. (A)** Correlation analysis of differential metabolites and differential transcripts. The horizontal axis represents differential metabolites, and the vertical axis represents differential transcripts, uric acid is marked with red triangles. **(B)** The changes in the mRNA expression levels of SLC22A23, SLC13A3 and SLC17A5 in GKOs after infection with GAstV. **(C)** The mRNA expression levels of SLC22A23, SLC13A3 and SLC17A5 in the kidneys of geese with varying degrees of gout due to infection with GAstV were changed.

To clarify which differentially expressed transcripts are correlated with uric acid, the core metabolite focused on in this study, correlation analysis was performed. The correlation heatmap (Fig. 6**A**) presented the top-ranked metabolites and their corresponding transcripts, among which eleven transcripts were significantly correlated with uric acid. According to published literature, three genes, namely SLC22A23, SLC17A5 and SLC13A3, are presumed to participate in renal uric acid excretion, all showing positive correlation coefficients with uric acid. All three molecules are solute carrier proteins expressed in kidney proximal tubules, which may suppress uric acid excretion via modulating organic anion transport and consequently elevate uric acid levels. Further validation in GKOs revealed that the mRNA expression levels of SLC22A23 and SLC17A5 were persistently and significantly upregulated throughout the infection period, whereas SLC13A3 exhibited a trend of initial downregulation followed by subsequent upregulation (Fig. 6**B**). Consistent results were further verified in clinical symptoms. The mRNA abundances of these three genes were markedly elevated in tissues with more severe gout symptoms, highlighting their vital functional significance (Fig. 6**C**). The molecular mechanism by which GAstV regulates the above molecules to trigger kidney metabolic disorders and elevate uric acid levels remains to be further elucidated in our ongoing investigations.

## Discussion

The highly lethal gout disease caused by GAstV in goslings poses a huge challenge to disease prevention and control in China's goose industry (Li et al., 2025; Liu et al., 2025). To address this challenge, it is necessary to progressively advance and refine our theoretical understanding of its pathogenic mechanisms. Owing to the limitations of traditional GAstV infection models, the feasibility, convenience, and scientific nature of in-depth exploration of its pathogenic mechanism are greatly restricted. Based on this, this project considered whether it is possible to establish an organoid model that can be infected with GAstV and simulate the tissue structure and physiological functions of the body. Because one of the main target organs of GAstV is the kidney, establishing goose kidney organoids is the first challenge that must be overcome.

As it was not clear how many days old goose embryo kidneys were most suitable for the isolation of primary GKOs, comparisons were made between 20-day-old (the optimal age for preparing GEK cells), 28-day-old, and 1-day-old goslings. The results showed that when preparing primary GKOs using 28-day-old goose embryo kidneys, growth status and speed were optimal. This might be because the kidneys begin to develop and form at 20 days of age. Although the structures of the glomeruli and renal tubules have already appeared, they are not yet fully developed. At this stage, they are unsuitable for the isolation and culture of primary kidney organoids. However, at 28 days of age, the kidneys were completely developed, the structures of the glomeruli and renal tubules were more complete, and the function of the chicken embryo kidneys was also more robust. However, they did not stop differentiating. Therefore, they are more suitable for *in vitro* culture after the isolation of GKOs. Immunofluorescence staining using markers for various cell lineages in the kidneys and HE staining confirmed that the prepared organoids were indeed renal organoids. Furthermore, immunohistochemical analysis and RT-qPCR confirmed that GAstV could infect and replicate within GKOs.

Double-label immunofluorescence staining analysis using protein markers of the renal cell lineage and the GAstV VP27 protein clearly determined the infection location of the virus in GKOs. GAstV colonizes the renal progenitor cells, pluripotent stem cells, collecting ducts, and distal regions. This indicated that GAstV can infect various cell lineages of GKOs and cause significant damage, resulting in the disruption of their normal metabolic functions. When the renal tubules, which function in uric acid secretion and transport, or the collecting ducts, which function in uric acid reabsorption and transport, are damaged, uric acid excretion can be obstructed, resulting in its accumulation and the occurrence of high uric acid levels (Eckenstaler and Benndorf, 2021; Ramos and Goldfarb, 2022). Furthermore, we compared the location of GAstV infection in the kidney tissues of geese, and the results were consistent with those of GKOs. It can infect multiple cell lineages in the kidneys. This highlights the reliability and scientific nature of the GKOs. This study also provided supporting evidence that, after GAstV infection in GKOs, the levels of uric acid, creatinine, and uric acid nitrogen significantly increased. However, microscopic observations revealed that GAstV can cause significant lesions in smaller, immature GKOs, while causing limited damage to larger, mature organoids. This may explain why the incidence rate in goslings is higher than that in adult geese during clinical breeding (Liu et al., 2025).

The GAstV infection model established in this study was superior to that of conventional 2D cells. We found that a high GAstV titer rapidly increased uric acid levels in GKOs. Even a lower-titer virus could cause high uric acid levels at 96 h after infection, which could simulate the pathogenic process of GAstV in goslings. However, in traditional infection models, such as LMH, GEK, and GEH cells, uric acid levels did not significantly increase after GAstV infection, which cannot simulate the pathological process in the body. Therefore, there is a great limitation in the exploration of its pathogenic mechanism. Additionally, conventional 2D cell structures are monotonous and cannot simulate the physiological functions of internal organs in the body. We also found that the replication level of GAstV in 2D cells was significantly lower than that in GKOs cells. This further highlights the limitations of traditional 2D cells. Based on the GKOs platform, it was further confirmed that GAstV is a key causative factor of gout in young geese. Thus, only GAstV can cause high uric acid levels, whereas TMUV and GRV cannot achieve the same effect. Furthermore, the GAstV load and uric acid levels in the kidneys of geese with severe gout were significantly higher than those of geese in the mild and asymptomatic groups. This finding aligns with the phenomenon that high-titer viruses can rapidly induce high uric acid levels in GKOs. In other words, the GKO infection model established by high-titer GAstV infection can recapitulate the renal tissue characteristics of geese with severe gout caused by clinical GAstV infection, suggesting that the efficient replication of GAstV may be a key factor contributing to gout in young geese.

Metabolomic analyses demonstrated that GAstV infection predominantly disturbs purine metabolism and its upstream and downstream pathways including one-carbon metabolism and amino acid metabolism. It facilitates uric acid production via synergistically modulating purine anabolism and catabolism, and also induces systemic redox disturbance and metabolic stress, thereby remodeling the microenvironment for uric acid metabolism and excretion. Transcriptomic data indicated that GAstV activates receptor-mediated inflammatory pathways and initiates innate immune responses. Inflammatory effects are further amplified through cell adhesion and calcium signaling pathways. Meanwhile, viral infection interferes with lipid and amino acid metabolism. These multi-layered synergistic effects collectively contribute to the development of hyperuricemia. Given that uric acid is the predominant cause of gout pathogenesis, we mainly focused on transcripts associated with uric acid metabolism and calculated the correlations between differential metabolites and transcripts. This strategy aimed to screen out core molecules regulated by GAstV that mediate uric acid elevation.

Among the 11 differentially expressed genes significantly correlated with uric acid levels, SLC17A5, SLC13A3 and SLC22A23 were selected as priority candidates for subsequent validation based on their functional characteristics, renal expression profiles and potential relevance to uric acid metabolism. All three genes encode solute carrier proteins with high renal expression. Specifically, SLC13A3 indirectly supports renal urate secretion by supplying energy substrates for urate transport; SLC17A5 is involved in maintaining tubular microenvironmental homeostasis via regulation of acid-base and anion balance; and SLC22A23 mediates organic anion transport in renal tubules (Bennett et al., 2011; Burckhardt et al., 2005; Donoghue et al., 2022; Engelhart et al., 2020; Reimer, 2013). Their reported biological functions show potential relevance to the core mechanism of impaired uric acid excretion induced by GAstV infection. In contrast, the remaining candidate genes are mainly involved in secondary pathological processes including blood coagulation, inflammatory injury, tissue structural remodeling, lipid metabolism and immune response, and show weaker relevance to pathways governing uric acid transport and excretion. Accordingly, these three transporter genes were prioritized as candidate molecules for exploring the mechanisms underlying virus-induced hyperuricemia.

Studies have revealed that SLC13A3 mediates renal tubular dicarboxylate transport and supplies energy substrates for canonical urate transporters (Breljak et al., 2016), serving as a crucial driving factor for renal urate efflux, and its functional abnormality directly hinders renal urate secretion (Dewulf et al., 2019; Kumari et al., 2023). As a member of the organic anion transport system, SLC22A23 participates in the transport of organic acids and anions in renal tubules. It may potentially affect renal uric acid excretion efficiency through crosstalk or competitive interactions with urate-related transport pathways, though direct evidence for its involvement in urate transport remains limited (Ekizoglu et al., 2018; Yee and Giacomini, 2021). SLC17A5 regulates acid-base balance and anion homeostasis within renal tubules, which is required to maintain the physiological microenvironment for normal urate transport. Dysregulated expression of this gene may indirectly interfere with tubular uric acid excretion by disrupting this microenvironmental balance (Sreedharan et al., 2010; Wolfenson et al., 2026). Nevertheless, the precise roles of these three molecules in GAstV-induced renal metabolic disturbance and subsequent uric acid elevation remain largely unclear, and their causal involvement requires further functional validation. Elucidating the potential regulatory mechanisms of these candidate transporters will be the core focus of our follow-up investigations.

In summary, we demonstrated that GKOs could be used to simulate the multicellular phenotype of the goose kidney, which is one of the main target organs of GAstV. Our results provide important insights into the association between GAstV infection and gout-related events and indicate that GKOs can serve as a unique model to define the complex relationship between GAstV and high uric acid levels. Using this organoid model, three pivotal molecules (SLC22A23, SLC17A5, SLC13A3) that are potentially regulated by GAstV to trigger uric acid elevation were screened out. Nevertheless, their specific molecular mechanisms remain to be further investigated. This has a profound effect on GAstV pathogenesis. GKOs also provide an ideal platform for screening GAstV antiviral drugs and can be used for the isolation and growth of previously nonreplicable viruses.

## Availability of data and materials

Datasets used or analyzed in this study are available by reasonable request from the corresponding author.

## Author Contribution

Yong Xiang Conceived the overall research idea, collected and sorted all experimental data, completed quantitative statistical analysis, designed the core research scheme, verified the reliability of all test results, drafted the full original manuscript, revised and polished the whole paper repeatedly, and applied for the research funding that supported this study. Linlin Li Collected and organized experimental datasets, conducted statistical analysis of research data, and carried out all relevant experimental research work. Chenggang Liu Responsible for data collection, collation and statistical analysis of experimental results. Junqin Zhang Performed statistical analysis on all collected research data. Yunzhen Huang Finished statistical analysis of experimental data and participated in designing the research framework and technical schemes. Qi Zhai Completed the experimental operations and relevant research investigations for this project. Ming Liao Put forward the core conception and research framework of this work. Minhua Sun Secured the financial funding supporting this research and supervised the overall progress of the whole study. Jiawen Dong Proposed the research concept, arranged and conducted experimental investigations, managed the daily administration of the research project, and supervised the whole research and manuscript preparation process.

## Disclosures

The authors declare that they have no known competing financial interests or personal relationships that could have appeared to influence the work reported in this paper.

## Appendix Supplementary materials

Image, application 1

Image, application 2

## Footnotes

Supplementary material associated with this article can be found, in the online version, at doi:10.1016/j.psj.2026.107500.
