# Supplementary material for: Goose kidney organoids: A new model for studying the metabolic mechanism of goose astrovirus-induced gout
Source: Poult Sci. 2026 Jul 27;105(11):107500. doi: 10.1016/j.psj.2026.107500 (PMC13521225; doi:10.1016/j.psj.2026.107500)
Supplement: Supplementary file 1 [file mmc1.docx]

**Supplementary Fig. S1. The effects of preparing GKOs using goose embryos of different ages. (A)** 20-day-old goose embryos; **(B)** 28-day-old goose embryos; **(C)** 1-day-old goslings. The picture shows the growth status of GKOs after one week of cultivation of primary kidney cells. Scale bars: 500 μm.

**Supplementary Fig. S2. Uric acid levels and GAstV viral load in kidney tissues with different degrees of gout.** ** p < 0.01, *** p < 0.001, **** p < 0.0001.

**Supplementary Fig. S3. Global metabolome changes induced by GAstV infection in GKOs. (A)** Number of differential metabolites in GAstV-infected group compared with control group. **(B)** Volcano plot: Correlation between significance and effect of differential metabolites. **(C)** Heatmap of differential metabolites. **(D)** Variable Importance in Projection plot. The higher the VIP score, the greater its contribution to distinguishing metabolic differences between the Control and GAstV groups. **(E)** KEGG enrichment bubble plot. **(F)** Quantification of differential metabolites (uric acid, creatinine and urea nitrogen).

**Supplementary Fig. S4. Global transcriptome changes induced by GAstV infection in GKOs. (A)** Number of differential transcripts in GAstV-infected group compared with control group. **(B)** Volcano plot: Correlation between significance and effect of differential transcripts. **(C)** GO enrichment analysis. **(D)** KEGG enrichment bubble plot.
