# Supplementary material for: Goose kidney organoids: A new model for studying the metabolic mechanism of goose astrovirus-induced gout
Source: Poult Sci. 2026 Jul 27;105(11):107500. doi: 10.1016/j.psj.2026.107500 (PMC13521225; doi:10.1016/j.psj.2026.107500)
Supplement: Supplementary file 2 [file mmc2.docx]

**Supplementary Table S1 Core pathway-related metabolites**

| Pathway | Metabolite |
| --- | --- |
| Purine metabolism | Xanthine(MN5229.neg);  Uric acid(MN6090.neg); Glutamine(MP3673.pos);  Inosinic acid(MN14827.neg); ADP(MN18478.neg);  Adenosine monophosphate(MP15106.pos); Adenosine(MP10365.pos);  Hypoxanthine(MN4338.neg);  Xanthosine(MN11956.neg);  Inosine(MN11212.neg);  Guanosine monophosphate(MN15574.neg) |
| Pantothenate and CoA biosynthesis | Coenzyme A(MN16504.neg); Pantetheine(MN11707.neg);  L-Valine(MP2020.pos);  L-Aspartic acid(MP2934.pos) |
| Arginine biosynthesis | Glutamic acid(MP3723.pos);  L-Arginine(MP5133.pos);  L-Aspartic acid(MP2934.pos); Glutamine(MP3673.pos) |
| One carbon pool by folate | Methionine(MP3811.pos);  Sarcosine(MP761.pos);  S-Adenosylhomocysteine(MP6063.pos); Adenosine(MP10365.pos); Choline(MP23617.pos) |
| D-Amino acid metabolism | Glutamine(MP3673.pos);  Glutamic acid(MP3723.pos);  2-Oxoarginine(MP5071.pos) |
| Glutathione metabolism | Glutamic acid(MP3723.pos);  Glutathione(MN13017.neg);  Oxidized glutathione(MN26609.neg); Cadaverine(MP611.pos) |
| Nitrogen metabolism | Glutamine(MP3673.pos);  Glutamic acid(MP3723.pos) |
| Arginine and proline metabolism | L-Arginine(MP5133.pos); Creatine(MP2814.pos);  4-Guanidinobutanoic acid(MP3614.pos); Glutamic acid(MP3723.pos) |
